# Rivaroxaban in Valvular Atrial Fibrillation — a Critical Appraisal of the INVICTUS Trial

**DOI:** 10.1007/s10557-023-07470-z

**Published:** 2023-05-25

**Authors:** Andreas Hammer, Alexander Niessner, Patrick Sulzgruber

**Affiliations:** Division of Cardiology, Department of Internal Medicine II, Medical University of Vienna, Waehringer Guertel 18-20, 1090 Vienna, Austria

**Keywords:** INVICTUS trial, Critical appraisal, Rivaroxaban, Valvular atrial fibrillation

## Abstract

**Purpose:**

The efficacy of factor Xa inhibitors in patients with atrial fibrillation (AF) and rheumatic heart disease (RHD) is unknown.

**Methods/Results:**

The objective of this article was to conduct a comprehensive evaluation of the INVICTUS trial, an open-label randomized controlled study that compared vitamin K antagonists (VKA) to rivaroxaban in patients with AF and RHD while also considering the existing evidence from literature in this particular area of research.

**Conclusion:**

The findings of the INVICTUS trial indicated that rivaroxaban was found to be inferior in efficacy to VKA. However, it is important to note that the primary outcome of the trial was driven by sudden death and death caused by mechanical pump failure. As a result, it is necessary to approach the data from this study with caution, and it would be inappropriate to draw parallel conclusions for other causes of valvular AF. Particularly, the perplexing issue of how rivaroxaban could have contributed to both pump failure and sudden cardiac death requires further explanation. Additional data regarding changes in heart failure medication and ventricular function would be essential for proper interpretation.

Several landmark trials investigated the efficacy of non-vitamin-K antagonists (NOACs) in patients with nonvalvular atrial fibrillation (AF). Overall, NOACs provided a better net-clinical benefit as compared to vitamin K antagonists (VKA) and thus became standard of care in the majority of patients with nonvalvular AF [1, 2]. However, a particular population of patients with rheumatic heart disease (RHD) was excluded from the majority of previous AF trials. To address this issue, S.J. Connolly et al. investigated the efficacy of rivaroxaban compared to VKA in patients with RHD within INVICTUS (INVestIgation of rheumatiC AF Treatment Using Vitamin K Antagonists) — an international open-label noninferiority randomized controlled trial [3]. Since RHD is especially prevalent in lower-income regions, patients were predominantly enrolled in Africa, south Asia, and Latin America. Inclusion criteria were the presence of AF and echocardiographically documented RHD, and one or more of the following: CHA_2_DS_2_-VASc score ≥ 2, mitral valve area ≤ 2 cm^2^, either left atrial spontaneous echo contrast or left atrial thrombus. Patients with mechanical heart valves, dual antiplatelet therapy, medication with dual strong inhibitors of CYP4A4 and P-glycoprotein, terminal renal insufficiency (eGRF < 15 ml/min/1.73m^2^), and pregnancy were excluded. The primary efficacy outcome was initially defined as stroke or systemic embolism and was later expanded to a composite of stroke, systemic embolism, myocardial infarction, and death from vascular or unknown causes. Safety outcome was predefined as major bleeding in accordance with the International Society of Thrombosis and Hemostasis. Follow up visits were conducted 30 days after randomization, following every 6 months for a total of 48 consecutive months.

In total, 4565 patients were included, predominantly young females (median 50.5 years; 72.3%), and followed over a mean time period of 3.1 years. Mitral stenosis with a valve area of ≤ 2.0cm^2^ was present in 81.9%, and the CHA_2_DS_2_-VASc score was 1.9. Of the participants, 52.8% were already on VKA therapy.

The authors found that oral anticoagulation (OAC) with rivaroxaban q.d. (20 to 15 mg according to renal function) was inferior to VKA in efficacy, with a trend to lower but non-significant overall major bleeding events in the rivaroxaban arm. Fatal bleedings were rare, but significantly higher in the VKA group. Efficacy-wise, a hazard ratio of 1.25 was reported; however, hazard proportion assumption was not met. Nevertheless, restricted mean survival time differences showed significant results in efficacy for VKA within an intention-to-treat analysis. Mortality rates within this relatively young population were exceedingly high with 22% referring to 994 deaths, among them 552 rivaroxaban-receiving patients. The risk for ischemic stroke was lower within the VKA group; however, the main outcome driver was sudden death and death due to mechanical pump failure which accounted for about 64.9% of all observed events. It remains controversial how rivaroxaban should have contributed to such worse outcome. Although there were significant differences in observed ischemic stroke rates, these might have been greatly influenced by higher therapy discontinuation in the rivaroxaban arm (23% *n* = 513 vs. 6% *n* = 135). Although, the most common reasons for permanent discontinuation of rivaroxaban were valve surgery-crossover (32%, *n* = 161) and patient decision/non-compliance (33%; *n* = 169). Notably, among all permanent rivaroxaban discontinuations (*n* = 513), a third was then switched to study VKA.

Additionally, VKA-treated patients had regular INR measurements at their local physician or cardiologist. VKAs were titrated to achieve an INR of 2.0–3.0; however, the desired INR levels were not reached in two-thirds of patients until the first year; subsequently, a stable INR was reached in about two-thirds within 2 years. Since, a benefit of VKA therapy was observed after 18 to 24 months of follow up, this might reflect better overall patient care and especially in the regard to heart failure (HF), given that in almost 40% of participants, HF was already established at baseline. There were no differences in prescribed HF medication substance classes observed; however, no data on dose adjustments during the course of the study were reported. Interestingly, hospitalization for HF (HHF) rates was nearly identical between the groups, although this outcome variable might not be feasible in development-country populations since the threshold for hospitalization varies substantially [4]. HHF/death-ratio shows considerable regional differences. While in low-income countries a ratio of up to 1:1 can be seen, wealthier nations with more sophisticated healthcare present with a HHF/death-ratio of roughly 4–3:1 [5–8].

In 43.6% of enrolled individuals, the thromboembolic risk was considerably low — indicated by a CHA_2_DS_2_-VASc score of ≤ 2 and ultimately an observed event rate of only 0.9% per year — which gives the impression that the preservation of ventricular function rather than stroke prevention needs to be aimed at this highly vulnerable young patient population. Consequently, it seems crucial to prioritize optimizing the established HF medication in order to improve cardiac function and decrease mortality and morbidity.

Further contributing to an overall low thromboembolic risk might have been the inclusion criterium of mitral valve stenosis with an opening area of ≥ 2 cm^2^. From the perspective of current guidelines, progressive rheumatic mitral valve stenosis is defined as a planimetered valve area of ≤ 1.5 cm^2^ [9]. In consideration of this relatively low thromboembolic risk, in the Kaplan Meier plots for mortality and stroke or systemic embolism can be recognized that the former curves diverge at month 20 whereas the latter substantially later at month 32. This might indicate that thromboembolic events had no considerable influence on mortality. Moreover, at that time, the slopes for ischemic events showed an abrupt incline that resulted in an elevated risk for the rivaroxaban arm. The authors themselves stated that the significantly higher mortality rates for rivaroxaban were almost entirely driven by higher rates of sudden cardiac death and death due to mechanical or pump failure [3]. However, this endpoint reaches beyond the therapeutic spectrum of anticoagulant agents.

Nonetheless, the investigators of INVICTUS addressed an important research question in a population that was previously neglected in randomized controlled trials. However, it is important to note that the application of these results might be highly influenced by the area of enrollment of the patient. Hence, data of the present trial have to be interpreted with caution, and no parallel conclusions for other etiologies of valvular AF should be drawn. Especially, the conundrum of how rivaroxaban should have contributed to both pump failure and sudden cardiac death remains to be explained. Additional data on changes in HF medication and ventricular function would be crucial for interpretation, only then can we decide whether VKAs are truly superior to rivaroxaban in patients with RHD and AF.

## Data Availability

Not applicable.
